# The Impact of Intimate Partner Violence on the Mental and Physical Health of Sexual and Gender Minorities: A Comprehensive Review of Quantitative Research

**DOI:** 10.1007/s10508-024-03023-z

**Published:** 2024-11-04

**Authors:** Mariana Rodrigues, Annaliese Neaman, Julia Ditzer, Anat Talmon

**Affiliations:** 1 Department of Psychology, Stanford University, Stanford, CA USA; 2 Department of Social & Behavioral Sciences, School of Global Public Health, New York University, New York, NY 10003 USA; 3 Faculty of Psychology, Clinical Child and Adolescent Psychology, Technische Universität Dresden, Dresden, Germany; 4 Paul Baerwald School of Social Work and Social Welfare, The Hebrew University of Jerusalem, Jerusalem, Israel

**Keywords:** Intimate partner violence, Sexual orientation, Gender identity, Adverse outcomes, Mental health

## Abstract

The aim of this review was to investigate the mental and physical health outcomes of intimate partner violence (IPV) victimization among sexual and gender minority (SGM) individuals. This study addressed an existing gap in research concerning IPV outcomes among SGM populations. A systematic review was conducted through PubMed, APA PsycInfo, APA PsycNet, and manual searches on Google Scholar using specific keywords. Inclusion criteria included peer-reviewed publications and quantitative studies specifically assessing the impacts of IPV among SGM individuals. A total of 35 studies meeting these criteria were included in the review. Our review showed significant associations between IPV victimization and adverse mental and physical health outcomes, including posttraumatic stress disorder, depression, anxiety, suicidal ideation and attempts, substance use, and risky sexual behaviors among SGM individuals. IPV victimization poses mental and physical health risks for SGM populations. Our findings highlight the need for comprehensive, tailored intervention and prevention efforts that consider the diverse needs of individuals with multiple minoritized identities in the context of IPV victimization.

**Supplementary Information:**

The online version contains supplementary material available at 10.1007/s10508-024-03023-z.

## Introduction

Intimate partner violence (IPV), as defined by the Centers for Disease Control and Prevention (CDC), encompasses various forms of abuse and aggression within romantic relationships and presents a significant and pervasive global public health concern (CDC, 2021; Garcia-Moreno et al., 2006). IPV manifests as episodes of violence perpetrated by current or former partners, including spouses, dating partners, or intimate partners (CDC, 2021). This violence encompasses a broad spectrum of aggressive behaviors, such as physical violence, sexual violence, stalking, financial exploitation, and psychological abuse (i.e., intense criticism, coercive control, and verbal harassment; CDC, 2021). Additionally, sexual and gender minority (SGM) individuals experience unique IPV tactics such as identity-specific abuse (i.e., abuse related to an individual’s sexual and/or gender minority identity (Scheer et al., 2019; Stults et al., 2023).^1^

Extensive research has illuminated the impact of IPV victimization on millions of individuals annually, transcending demographic categories such as age, gender, race, ethnicity, socioeconomic status, sexual orientation, and nationality (Devries et al., 2013). However, scant attention has been given to understanding the adverse consequences of IPV victimization among individuals with SGM identities. Therefore, this study seeks to investigate the impacts of IPV victimization among individuals who identify as having a sexual- and/or gender-minoritized identity. Our aim is to address the existing gap in IPV research concerning those with SGM identities and to highlight the need for inclusive interventions that target SGM populations.

### Violence Exposure Among Sexual and Gender Minorities

According to Whitton et al. (2019), SGM individuals are at greater risk for IPV than their cisgender heterosexual counterparts due to minority stressors. First introduced by Brooks (1981), minority stressors can be defined as stressors that (1) add to the daily stress experienced by the general population, necessitating additional adaptation strategies by individuals with stigmatized identities; (2) are chronic and rooted in the complex interplay between minoritized identities and systems of oppression; and (3) are socially constructed, stemming from structural processes beyond individual control (Meyer, 2003). In addition to experiencing IPV, SGM individuals often contend with additional stressors that merit consideration when examining the consequences of victimization. These stressors include but are not limited to, discrimination, victimization due to minority status, internalized stigma, transphobia, or homophobia (Meyer, 2003), all of which have been associated with increased levels of IPV perpetration and victimization (Badenes-Ribera et al., 2019; McCown, 2023; Sarno et al., 2023; Stephenson & Finneran, 2017; Whitton et al., 2024).

Previous studies have shed light on how minority stressors can contribute to the experience of SGM-specific victimization in an intimate relationship. For instance, Balsam and Szymanski (2005) found that adult lesbian and bisexual women often reported experiencing IPV tactics that involve leveraging the societal stigma of their identities to exert control (i.e., disclosing the victim’s SGM identity without their consent, shaming them for their identity, questioning their identity, and isolating them from their SGM support system). Additionally, research focused on IPV within the transgender community has found that transgender individuals may face increased IPV risk in part because perpetrators might leverage societal transphobia as a tool of power and control (Peitzmeier et al., 2021). Examples of transgender-specific IPV (T-IPV) include, but are not limited to, undermining the individual’s identity by misgendering them as well as forcing victims to engage in sexual relationships by threatening to disclose their transgender identities to their families (Guadalupe-Diaz, 2017; Peitzmeier et al., 2020). Thus, considering how perpetrators might leverage various systems of oppression and societal norms against their SGM partner, it is imperative to conduct research focused solely on the unique experiences and impacts of IPV among SMG individuals. Additionally, it is crucial to recognize that SGM individuals are not a monolithic group. Research must consider how diverse sexual orientations and gender identities intersect with various systems of oppression to understand the unique experiences and needs of these individuals.

While IPV research has historically focused on cisgender heterosexual women, recent studies have embraced an intersectional perspective to explore how various minority identities intersect with systems of oppression concerning IPV. These studies have identified, for instance, differences in IPV outcomes based on immigration status, cultural background, and socioeconomic factors (Sabina et al., 2014; Silva-Martínez, 2017). Much of the work, however, still remains confined to the experiences of cisgender heterosexual women and examined through a cisgender heteronormative lens, while SGM individuals are still largely overlooked. Additionally, SGM-IPV literature has primarily focused on documenting differences in IPV prevalence and experiences between SGM and cisgender heterosexual individuals (Peitzmeier et al., 2020).

While some scholars have highlighted the need to use comprehensive approaches to IPV research (Brooks et al., 2021; Burgess-Proctor, 2006; Subriana-Malaret et al., 2019) studies focused on SGM individuals and examining how identities such as sexual orientation and gender identity specifically impact IPV experiences remain scarce. Veldhuis et al. (2024) have recently emphasized the importance of centering the lived experiences of minoritized individuals, rather than comparing them to majority groups. While Veldhuis et al. recognize that such comparisons can offer insights, they argue that this approach implies a group's experiences are only meaningful when compared to a dominant group (Bowleg, 2021).

In line with this perspective, the current review aims to focus on the experiences of IPV among SGM populations without drawing comparisons from their cisgender heterosexual counterparts. This review examines the specific mental and physical health outcomes of IPV victimization among SGM individuals within the context of their sexual orientation and/or gender identities.

## Method

### Data Source and Search Strategy

The current systematic review followed the Preferred Reporting Items for Systematic Review and Meta-Analyses (PRISMA) statement (Page et al., 2021). Two reviewers conducted one systematic search in August 2023 and a second one in August 2024 through PubMed, APA PsycInfo, APA PsycNet, and manual searches on Google Scholar. The search strategy included the keywords “Intimate Partner Violence” and “Sexual and Gender Minorities,” which were used to find MeSH terms and ultimately conduct the searches in the abovementioned platforms^2^. A table of the search strategy and terms used is provided in Appendix S1.

### Inclusion and Exclusion Criteria

The following inclusion criteria were applied to select the articles: (1) in the English language, (2) published in peer-reviewed journals, (3) quantitative studies, and (4) focused on the assessment of the outcomes of IPV among SGM individuals.

The studies that did not match the inclusion criteria above were excluded. In addition, the following exclusion criteria were applied: (1) studies about IPV in which the methods or results did not differentiate between IPV among SGM and cisgender, heterosexual individuals, (2) studies that assessed the outcomes of IPV as a joint factor with other stressors not directly experienced due to minority identity (e.g., the COVID-19 pandemic, childhood maltreatment, peer victimization, bullying, etc.), (3) studies that assessed IPV’s outcomes among SGM individuals but did not differentiate between perpetration- and victimization-related impacts, or (4) if the sample size included less than 50% of participants belonging to a sexual and/or gender minority group, given our primary focus on SGM instead of comparison with their cisgender heterosexual counterparts.

### Data Extraction Process

Overall, the search resulted in 12,376 articles imported into Covidence Software. Of these, 174 papers were selected for full-text review after the title and abstract screening process, and 35 papers remained applicable to the criteria and were included in the systematic review.

**Fig. 1:**
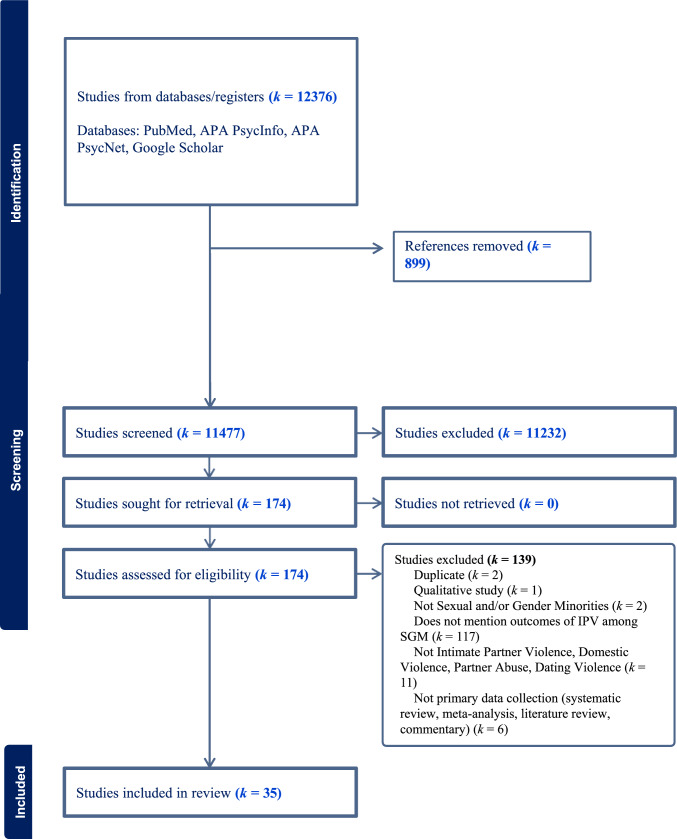
PRISMA flowchart

Any disagreement regarding the data extraction stage was discussed between the reviewers to obtain a unanimous agreement. See the flowchart of the selection procedure (Fig. 1).

### Quality Assessment

The methodological quality of the included studies was assessed by the first two authors (MR and AN) using the NIH Quality Assessment Tool for Observational Cohort and Cross-Sectional Studies. This tool comprises 14 criteria that assess internal validity, including risk of selection bias, information bias, measurement bias, and confounding. The evaluators selected “Yes,” “No,” or “Not Reported” (RC), “Not applicable” (NA), or “Cannot Determine” (CD) in the NIH tool. An overall assessment of each study was generated based on the number of evaluators’ responses. Results of the quality assessments of individual studies are shown below (Table 1).

**Table 1:** NIH Quality Assessment Tool for Observational Cohort and Cross-Sectional Studies

Study	Q1	Q2	Q3	Q4	Q5	Q6	Q7	Q8	Q9	Q10	Q11	Q12	Q13	Q14
Becerra et al. (2021)	Y	Y	NR	Y	Y	N	N	Y	N	N	Y	NA	NA	Y
Beymer et al. (2017)	Y	Y	NR	Y	Y	N	N	Y	N	N	Y	NA	NA	Y
Braksmajer et al. (2019)	Y	Y	NR	Y	Y	N	N	Y	Y	N	Y	NA	NA	Y
Bukowski et al. (2019)	Y	Y	NR	Y	Y	N	N	N	N	N	Y	NA	NA	Y
Chen et al. (2019)	Y	Y	NR	Y	N	N	N	Y	Y	N	Y	NA	NA	Y
Culbreth et al. (2023)	Y	Y	NR	Y	Y	N	N	Y	Y	N	Y	NA	NA	Y
Davis et al. (2022)	Y	Y	Y	Y	Y	N	N	Y	Y	N	Y	NA	NA	Y
Duncan et al. (2018)	Y	Y	Y	Y	N	N	N	Y	Y	N	Y	NA	NA	Y
Edwards et al. (2021)	Y	Y	NR	Y	Y	N	N	Y	Y	N	Y	NA	NA	Y
Goldberg-Looney et al. (2016)	Y	Y	NR	T	T	N	N	Y	Y	N	Y	NA	NA	N
Henry et al. (2021)	Y	Y	NR	Y	N	N	N	Y	Y	N	Y	NA	NA	Y
Hillman (2022)	Y	Y	Y	Y	Y	N	N	Y	Y	N	Y	NA	NA	Y
Houston and McKirnan (2007)	Y	Y	NR	Y	N	N	N	Y	N	N	Y	NA	NA	Y
McDowell et al. (2019)	Y	Y	NR	Y	Y	N	N	N	N	N	Y	NA	NA	Y
Metheny et al. (2024)	Y	Y	NR	Y	Y	N	N	Y	Y	N	Y	NA	NA	Y
Miller et al. (2024)	Y	Y	NR	Y	N	Y	Y	Y	Y	Y	Y	NA	NR	Y
Miltz et al. (2019)	Y	Y	Y	Y	Y	N	Y	Y	N	Y	Y	NA	N	Y
Pantalone et al. (2012)	Y	Y	Y	Y	N	N	N	Y	Y	N	Y	NA	NA	Y
Passaro et al. (2019)	Y	Y	NR	Y	Y	N	N	Y	N	N	Y	NA	NA	Y
Peitzmeier et al. (2021)	Y	Y	Y	Y	Y	N	N	Y	Y	N	Y	NA	NA	Y
Peng et al. (2020)	Y	Y	Y	Y	N	N	N	Y	Y	N	Y	NA	NA	Y
Reisner et al. (2013)	Y	Y	Y	Y	Y	N	N	N	N	N	Y	NA	NA	Y
Reuter et al. (2017)	Y	Y	NR	Y	Y	Y	Y	Y	Y	Y	Y	NA	Y	N
Scheer and Mereish (2021)	Y	Y	NR	Y	Y	N	N	Y	Y	N	Y	NA	NA	Y
Stults et al. (2015)	Y	Y	Y	Y	Y	N	N	Y	N	N	Y	NA	NA	Y
Stults et al. (2023)	Y	Y	NR	Y	N	N	N	Y	Y	N	Y	NA	NA	Y
Taber et al. (2023)	Y	Y	Y	Y	Y	N	N	Y	Y	N	Y	NA	NA	Y
Whitton et al. (2019)	Y	Y	NR	Y	N	Y	Y	Y	Y	Y	Y	NA	Y	Y
Wong et al. (2010)	Y	Y	NR	Y	N	N	N	Y	Y	N	Y	NA	NA	Y
Wirtz et al. (2022)	Y	Y	NR	Y	Y	N	N	Y	N	N	Y	NA	NA	Y
Woulfe and Goodman (2020)	Y	Y	Y	Y	Y	N	N	Y	Y	N	Y	NA	Y	Y
Xavier Hall et al. (2022)	Y	Y	NR	Y	Y	Y	Y	Y	N	N	Y	NA	Y	Y
Xu et al. (2022)	Y	Y	Y	Y	Y	N	N	Y	Y	N	Y	NA	NA	Y
Yu et al. (2023)	Y	Y	Y	Y	N	N	N	Y	Y	N	Y	NA	NA	Y
Zhu et al. (2021)	Y	Y	Y	Y	N	N	N	Y	Y	N	Y	NA	NA	Y

NR = Not Recorded. NA = Not applicable. Q1: Was the research question or objective in this paper clearly stated? Q2: Was the study population clearly specified and defined? Q3: Was the participation rate of eligible persons at least 50%? Q4: Were all the subjects selected or recruited from the same or similar populations (including the same time period)? Were inclusion and exclusion criteria for being in the study prespecified and applied uniformly to all participants? Q5: Was a sample size justification, power description, or variance and effects estimates provided? Q6: For the analyses in this paper, were the exposure(s) of interest measured prior to the outcomes(s) being measured? Q7: Was the timeframe sufficient so that one could reasonably expect to see an association between exposure and outcome if it existed? Q8: For exposures that can vary in amount or level, did the study examine different levels of exposure as related to the outcome (e.g., categories of exposure, or exposure measured as a continuous variable)? Q9: Were the exposure measures (independent variables) clearly defined, valid, reliable, and implemented consistently across all study participants? Q10: Was the exposure(s) assessed more than once over time? Q11: Were the outcome measures (dependent variables) clearly defined, valid, reliable, and implemented consistently across all study participants? Q12: Were the outcome assessors blinded to the exposure status of participants? Q13: Was the loss to follow-up after baseline 20% or less? Q14: Were key potential confounding variables measured and adjusted statistically for their impact on the relationship between exposure(s) and outcome (s)?

## Results

Thirty-five studies were included in the current systematic review. Of these, 27 studies were conducted in the USA. Four studies were conducted in China, one in England, one in Peru, one in Guatemala, and one in Taiwan. The majority of the studies were published in the last 5 years. One study was published each year in years 2007, 2010, 2012, 2013, 2015, 2016, and 2018. Two studies were published in 2017 and 2024, four studies were published in 2020 and 2022, five in 2019 and 2023, and six in 2021. The combined sample size of the 35 studies is 52,612.

### Intimate Partner Violence-Related Outcomes

#### Mental Health Outcomes

##### Posttraumatic Stress Disorder (PTSD)

The association between IPV victimization and PTSD was evident across multiple studies, highlighting a significant risk for SGM individuals. For instance, Taber et al. (2023) found that experiencing identity-specific IPV was associated with a significantly higher score on the Posttraumatic Stress Disorder Checklist (PCL-6; Han et al., 2016) (*p* < 0.01). Both the total and direct relationships of T-IPV and PTSD, as well as between identity abuse and PTSD, were significant, with confidence intervals indicating robust findings (95% CI).

Stults et al. (2015) demonstrated that, in a sample of young men who have sex with men, lifetime IPV victimization was significantly correlated with PTSD symptoms (*r* = 0.27, *p* < 0.01). Similarly, McDowell et al. (2019) found that lifetime IPV victimization (referent = no IPV; aOR = 3.08; 95% CI = 1.26, 7.53; *p* = 0.01) was significantly associated with increased odds of PTSD compared to individuals who had not experienced IPV. Among transgender women, Peitzmeier et al. (2021) reported that lifetime T-IPV was significantly associated with a 50% increased risk of PTSD (APR = 1.50, 95% CI = 1.31, 1.72).

Additionally, Woulfe and Goodman (2020) identified that identity abuse accounted for an additional 1% of the variance in PTSD symptoms after controlling for other forms of IPV and demographic variables (*r* = 0.11, *p* < 0.01). among lesbian, gay, bisexual, transgender, and queer individuals.

Stults et al. (2023) further illustrated that in the second step of a hierarchical linear regression model, the addition of lifetime IPV variables resulted in a statistically significant change in the variance accounted for by the model (ΔR^2^ = 0.12, *F*(5, 156) = 2.28, *p* = 0.006). Specifically, among transgender and gender-diverse young adults, both younger age (B = − 0.38, *p* = 0.016) and lifetime intimate abuse (IA) (B = 3.18, *p* = 0.009) were significantly associated with higher PTSD scores. In step three, while the addition of past-year IPV variables did not result in a significant change in the variance explained by the model, younger age (B = − 0.40, *p* = 0.012) and lifetime IA (B = 3.48, *p* = 0.011) continued to show significant associations with increased PTSD scores.

### Depression

IPV victimization has a significant association with depression across multiple studies, underscoring its profound impact on mental health among SGM individuals. For instance, Taber et al. (2023) reported that identity-specific IPV was linked to elevated scores on the PHQ-9 among transgender and gender nonconforming young adults, with significant total and direct relationships between transgender-related IPV and depression, as well as identity abuse (*p* < 0.01). Woulfe and Goodman (2020) supported this by demonstrating that identity abuse exposure accounted for an additional 1% of the variance in depression scores, while significant semi-partial correlations with depression symptoms (SR^2^ = 0.13; *p* < 0.01) were observed for both identity and physical abuse.

Furthermore, Stults et al. (2015) found a correlation between lifetime IPV victimization and depression symptoms (*r* = 0.23, *p* < 0.01) among Men Who Have Sex With Men (MSM). This aligns with Reuter et al. (2017), who highlighted that physical IPV victimization predicted higher levels of depression (*t* = 2.42, *p* = 0.02) among LGBT young adults.

Specific and diverse populations also show heightened vulnerability. For example, Bukowski et al. (2019) identified a 36% increased likelihood of depressive symptoms among Black transgender women experiencing past-year IPV (IRR = 1.36, 95% CI = 1.23, 1.50). Henry et al. (2021) further illustrated the complexity of these associations, finding significant links between psychological abuse (*r* = 0.245, *p* = 0.05), sexual abuse (*r* = 0.227, *p* = 0.05), and assault with injury (*r* = 0.270, *p* = 0.05) and depression among transgender and gender nonconforming adults.

Wirtz et al. (2022) reported that recent IPV victimization was associated with increased depressive symptoms among Black gay and bisexual men, even after controlling for confounders (adjPrR: 2.36; 95% CI: 1.61, 3.47). Miltz et al. (2019) observed that IPV victimization led to depressive symptomatology nearly three times higher than in non-victims (OR = 2.57, 95% CI = 1.71, 3.86; OR = 2.93, 95% CI = 1.71, 3.86) among gay and bisexual men.

Davis et al. (2022) further expanded on these findings by showing that various forms of lifetime IPV, including emotional IPV (OR = 5.14, 95% CI = 2.35, 11.27) and multiple forms of recent IPV (OR = 3.83, 95% CI = 1.54, 9.47), were significantly associated with depression among MSM. Notably, MSM living with HIV who experienced IPV had significantly higher odds of depression (OR = 2.02, 95% CI = 1.02, 3.99). Pantalone et al. (2012) corroborated this, finding that men reporting past-year physical partner abuse exhibited higher levels of depressive symptoms (26.2 vs. 18.4; *t*(168) =  − 3.2, *p* < 0.01).

Yu et al. (2023) also found that MSM living with HIV who had experienced any type of IPV were more likely to report depressive symptoms (aOR = 3.83; 95% CI: 2.09–7.02). Finally, Stults et al. (2023) noted that lifetime IPV significantly increased the variance explained in depression scores among transgender and gender-diverse young adults (ΔR^2^ = 0.15, *F*(5, 155) = 7.10, *p* < 0.001), with both lifetime identity abuse and past-year T-IPV showing significant associations (B = 3.37, *p* = 0.012; B = 5.66, *p* = 0.010). Similarly, Peng et al. (2020) found a significant positive correlation between experiences of various forms of IPV and depression in MSM (*r* = 0.206, *p* < 0.01).

### Anxiety

The association between IPV victimization and anxiety among SGM individuals is evident across multiple studies, demonstrating a significant impact on mental health. For example, Taber et al. (2023) found that any form of identity-specific IPV was linked to higher scores on the General Anxiety Disorder Scale (GAD-7; *p* < 0.01) among transgender and gender nonconforming young adults, emphasizing the role of identity abuse in exacerbating anxiety.

Reuter et al. (2017) further illustrated this connection, revealing that verbal IPV victimization was associated with increased anxiety levels one year later (*t* = 2.04, *p* = 0.04) among LGBT young adults. Similarly, Henry et al. (2021) identified strong correlations between various forms of IPV and anxiety symptoms among transgender and gender nonconforming adults, reporting effect sizes of *r* = 0.396 (psychological abuse), *r* = 0.45 (sexual abuse), *r* = 0.45 (physical abuse), and *r* = 0.46 (assault with injury), all with *p* < 0.01.

One study found that any form of lifetime IPV was significantly associated anxiety (OR = 2.10, 95% CI = 1.29, 3.40), with recent emotional IPV showing an even stronger association (OR = 5.08, 95% CI = 2.31, 11.20) (Davis et al., 2022). Pantalone et al. (2012) highlighted that men reporting past-year physical partner abuse had higher average anxiety scores (2.4 vs. 1.8 on the STPI; *t*(168) =  − 4.1, *p* < 0.001) compared to those not reporting such abuse.

Additionally, Yu et al. (2023) demonstrated that MSM living with HIV who experienced any type of IPV were significantly more likely to report anxiety symptoms (aOR = 2.27; 95% CI: 1.19–4.35) than those who did not experience IPV. Stults et al. (2023) reinforced these findings, showing that adding lifetime IPV variables in a hierarchical linear regression model significantly increased the variance explained in anxiety scores (ΔR^2^ = 0.12, *F*(5, 156) = 5.11, *p* = 0.001).

Notably, lifetime intimate abuse was significantly associated with higher anxiety scores (B = 2.89, *p* = 0.043).

### Suicidal Ideation and Attempts

The relationship between IPV victimization and suicidal ideation is supported by multiple studies highlighting a concerning trend among affected individuals. Xu et al. (2022) reported that transgender women who experienced both physical and verbal IPV in their lifetime had significantly increased odds of reporting suicidal ideation, with odds ratios of 2.58 (95% CI = 1.16–5.72) for one model and 2.72 (95% CI = 1.33–5.55) for the other.

In addition, Becerra et al. (2021) found that Asian-American transgender adults who reported experiencing romantic or sexual partner abuse exhibited markedly higher rates of suicidal thoughts (88.3% vs. 76.5%) and attempts (53.2% vs. 33.3%) compared to those without such experiences. These findings illustrate the critical intersection of identity and IPV in exacerbating suicide risk.

Additionally, Pantalone et al. (2012) found that men who reported past-year physical partner abuse had significantly higher suicidal ideation scores (1.0 vs. 0.6; *t*(168) = -2.5, *p* = 0.01) on the Passive Suicidal Behavior subscale of the Harkavy Asnis Suicide Survey (HASS) when compared to those not reporting abuse. Further emphasizing this connection, Yu et al. (2023) found that MSM living with HIV who had experienced any type of IPV—whether emotional, physical, or threats of identity exposure—were more likely to report suicidal ideation (aOR = 3.78; 95% CI: 2.11–6.80) than those who did not experience IPV.

Lastly, Hillman (2022) highlighted that among transgender adults aged 50 years or older, those who experienced any type of lifetime IPV had nearly double the odds of ever making a suicide attempt (AOR = 1.98; 95% CI = 1.66–2.46, *p* < 0.01) compared to those who did not experience IPV.

### Psychological Distress

IPV victimization was significantly associated with psychological distress (i.e., scoring 13 or higher on the Kessler-6 scale) across several studies. Becerra et al. (2021) found that serious psychological distress was notably higher among Asian-American transgender adults who reported experiencing romantic or sexual partner abuse, with rates of 45.3% compared to 25.5% among those without such experiences. Similarly, Peitzmeier et al. (2021) reported that lifetime experiences of T-IPV were linked to a 32% increased risk of serious psychological distress, with an adjusted prevalence ratio of 1.32 (95% CI = 1.13, 1.53) among transgender women.

In a study involving sexual minority youth, Whitton et al. (2019) found that experiences of sexual IPV were predictive of higher levels of psychological distress, with a correlation coefficient of *r* = 0.32 (*p* < 0.01). Furthermore, Hillman (2022) highlighted that among transgender adults aged 50 years and older, those who had experienced any form of lifetime IPV reported higher odds of severe psychological distress, as assessed by six items regarding feelings of depression, nervousness, hopelessness, fatigue, and worthlessness in the past month.

Specifically, the adjusted odds ratio was 1.32 (95% CI = 1.04–1.66, *p* < 0.01) compared to those who had not experienced IPV.

### Maladaptive Coping Styles

IPV victimization was significantly associated with maladaptive coping styles, characterized by self-distraction, denial, venting, substance use, behavioral disengagement, and self-blame. In a study by Goldberg-Looney et al. (2016) focusing on sexual minority men, psychological abuse was found to be positively associated with denial (*r* = 0.24, *p* = 0.05) and behavioral disengagement (*r* = 0.28, *p* < 0.01). Additionally, sexual abuse correlated with several maladaptive coping strategies, including self-distraction (*r* = -0.14, *p* < 0.01), denial (*r* = 0.25, *p* = 0.05), and behavioral disengagement (*r* = 0.48,* p* < 0.01). Physical abuse also demonstrated a significant relationship with denial (*r* = 0.21, p = 0.05) and behavioral disengagement (*r* = 0.40, *p* < 0.01). Furthermore, IPV victimization resulting in injury was significantly linked to both denial (*r* = 0.28, *p* < 0.01) and behavioral disengagement (*r* = 0.46, p < 0.01).

In addition, Pantalone et al. (2012) found that men reporting past-year physical partner abuse were more likely to exhibit avoidant coping styles, with average scores of 1.3 compared to 0.9 among those not reporting such abuse (*t*(168) =  − 3.9, *p* < 0.001).

### Risk Behaviors

#### Substance Use

Several studies illustrate the strong link between IPV and alcohol use. Edwards et al. (2021) reported that sexual IPV victimization was significantly associated with hazardous drinking among LGBTQ+ college students (*r* = 0.23, *p* < 0.05). Metheny et al. (2024) found that IPV victimization was strongly associated with future alcohol use, with both linear and quadratic terms significantly predicting AUDIT scores (B: 0.26, 95% CI: 0.14, 0.38), particularly noting that sexual IPV had the strongest association (B: 1.47, 95% CI: 0.39, 2.55). Wirtz et al. (2022) reported that recent IPV was linked to hazardous alcohol use in Black gay and bisexual men (adjPrR: 1.93; 95% CI: 1.42, 2.61), as well as emphasized the compound effects of IPV and substance use on depressive symptomatology, where the joint effect resulted in a twofold increase in depression symptoms (adj. PrR: 2.10, 95% CI: 1.51, 2.91).

In addition to alcohol, IPV is associated with increased use of marijuana and other illicit drugs. Whitton et al. (2019) found that both physical and sexual IPV were linked to increased marijuana use in a longitudinal study of sexual minority men (ERR = 1.21, 1.3) and elevated odds of drug use at subsequent study waves (ORs = 1.27, 1.21). Reisner et al. (2013) highlighted that IPV was strongly associated with substance misuse histories for individuals assigned male and female at birth, noting that IPV explained a substantial portion of the substance misuse disparity among gay and lesbian individuals (PEE = 38.91, *p* = 0.09).

Specific substance use behaviors have been documented among individuals experiencing IPV. Xavier Hall et al. (2022) found that IPV victimization was significantly correlated with alcohol problems (IRR = 1.62, 95% CI = 1.31, 2.00), cannabis issues (IRR = 1.55, 95% CI = 1.18, 1.93), and elevated odds for cocaine (OR = 2.87, 95% CI = 1.55, 4.99) and methamphetamine use (OR = 5.21, 95% CI = 1.86, 14.62) among sexual and gender minority youth, relative to those who experienced “low lifetime victimization” after adjusting for demographic characteristics. Passaro et al. (2020) indicated that MSM and transgender women reporting psychological IPV were more likely to meet the criteria for an alcohol use disorder (78.6% vs. 55.2% MSM; 100% vs. 62.8% TW; *p* < 0.05). Chen et al. (2019) reported that, in MSM living with HIV, experiences of IPV significantly predicted alcohol use (odds of any alcohol use increased by 100%) and other drug use (increased by 25%) with each unit increase in IPV (*p* < 0.05).

Miller et al. (2024) found that stimulant use was associated with nearly twice the odds of experiencing IPV among MSM (aOR = 1.85; 95% CI [1.35, 2.53]), specifically for physical IPV (aOR = 2.01; 95% CI [1.43, 2.81]). Pantalone et al. (2012) demonstrated that individuals reporting past-year physical partner abuse were more likely to use methamphetamine (46.9% vs. 25.2%) and powder cocaine (0.6% vs. 0.1%) in the past year (*p* < 0.001). Wong et al. (2010) found that experiencing physical violence was significantly associated with recent drug use (OR = 2.13; CI [1.39–3.26]; *p* ≤ 0.001), with specific increased odds for drug use associated with physical IPV (AOR = 2.01; CI [1.24–3.27]; *p* ≤ 0.01).

Moreover, studies emphasize the negative health behaviors associated with IPV victimization. Hillman (2022) indicated that transgender adults aged 50 and older with any lifetime IPV experience had increased odds of engaging in negative health behaviors, such as smoking (OR: 1.36, *p* < 0.01) and drug use (OR: 1.42, *p* < 0.01). Houston et al. (2007) found higher rates of substance use among gay and bisexual men who experienced IPV, reporting significant odds ratios for both monthly alcohol intoxication (OR = 1.43; 95% CI: 1.04–1.96) and substance use problems (OR = 1.84; 95% CI: 1.24–2.73).

Culbreth et al. (2023) found that IPV victimization was linked to tobacco use, in both bivariate (OR: 2.22; 95% CI: 1.17, 4.24) and multivariate analyses (OR: 2.14; 95% CI: 1.07, 4.28). Scheer and Mereish (2021) noted that physical abuse was related to illicit substance use among sexual and gender minority youth (*r* = 0.17, *p* < 0.05). Duncan et al. (2018) revealed a significant association between IPV victimization and illicit drug use, with those reporting IPV being 1.78 times more likely to engage in illicit drug use, noting that specific IPV types, such as physical and sexual IPV, were associated with higher odds of substance abuse in the past 30 days (ORs = 2.08 and 3.36, respectively).

Lastly, Peitzmeier et al. (2021) found that lifetime T-IPV was significantly associated with an increased risk of alcohol use disorder (APR = 1.18, 95% CI = 0.98, 1.42) and drug use disorder (APR = 1.20, 95% CI = 0.97, 1.48) among transgender women.

### Sexual Behaviors

IPV victimization significantly correlates with risky sexual behaviors among LGBTQ+ individuals, highlighting the adverse effects of violence on sexual health. Multiple studies illustrate this connection, revealing various forms of IPV linked to increased sexual risk.

Reuter et al. (2017) reported that verbal IPV was associated with higher rates of condomless sex acts (*t* = 2.29, *p* = 0.02) among LGBT adults, suggesting that even non-physical forms of IPV can elevate sexual risk. This is echoed by Braksmajer et al. (2020), who found that MSM experience emotional IPV (b = -0.74, 95% CI = -1.28, -0.20), monitoring (b = -0.07, 95% CI = -1.24, -0.14), or forced sex (b = -1.05, 95% CI = -1.93, -0.17) were less likely to utilize PrEP, indicating that IPV may hinder access to critical preventive measures.

Furthermore, Zhu et al. (2021) provided insights into the types of IPV influencing sexual health behaviors, finding that sexual (ORa = 2.11, 95% CI: 1.16, 3.85), control (ORa = 1.94, 95% CI: 1.00, 3.76), and emotional IPV victimization (ORa = 1.71, 95% CI: 1.02, 2.87) were positively associated with inconsistent condom use with regular partners. This was compounded by findings that controlling IPV victimization (ORa = 2.39, 95% CI: 1.011, 5.66) correlated with inconsistent condom use with casual partners, reinforcing the notion that IPV not only influences immediate sexual behaviors but also impacts broader sexual health risks, such as having multiple partners. Notably, any IPV victimization (ORa = 1.54, 95% CI: 1.02, 2.32) and sexual IPV victimization (ORa = 2.25, 95% CI: 1.30, 3.88) were linked to an increase in the number of regular sexual partners.

The link between IPV and HIV infection risk was underscored by Beymer et al. (2017), who reported that 20% of African-American MSM with a history of IPV tested HIV-positive compared to only 8% without such a history. This finding was supported by Passaro et al. (2020), which showed that MSM or transgender women who experienced IPV were more likely to engage in condomless receptive anal intercourse with one or more of their last three sexual partners (94.1% vs. 68.8%, *p* = 0.03). This connection between IPV and sexual risk is critical, as it underscores the need for comprehensive interventions addressing both violence and sexual health.

Similarly, Wirtz et al. (2022) focused on Black gay and bisexual men, revealing that recent IPV victimization was associated with a 1.4-fold increase in self-reported past 12-month STI diagnoses (adjPrR: 1.44; 95% CI: 1.08, 1.92). This study also found an inverse relationship between recent IPV and current PrEP use (adjPrR: 0.65; 95% CI: 0.38, 1.10), with physical IPV independently linked to reduced PrEP use (adjPrR: 0.35; 95% CI: 0.13, 0.90). Moreover, lifetime IPV victimization was significantly associated with lifetime ART medication interruptions among individuals living with HIV (adjPrR: 1.59; 95% CI: 1.25, 2.01), further emphasizing how IPV impacts both sexual behavior and health outcomes.

Yu et al. (2023) expanded this analysis by examining MSM living with HIV, finding that experiencing any type of IPV (including control, emotional, security threat, physical, sexual, and threat of public identity) was significantly correlated with sexual risk behavior (aOR = 2.02; 95% CI: 1.16 ~ 3.53) and poorer ART medication adherence (aOR = 2.63; 95% CI: 1.46 ~ 4.74) compared to those not experiencing IPV. This demonstrates that IPV not only exacerbates sexual risk behaviors but also complicates adherence to crucial health interventions.

Further reinforcing the link between IPV and risky sexual behaviors, Houston et al. (2007) reported that, among gay and bisexual men, IPV victimization was significantly associated with sexual risk, with 43.6% of IPV victims engaging in unprotected anal sex in the previous six months compared to 31.9% of those without IPV (OR = 1.61; 95% CI: 1.18–2.21, *p* < 0.05). Additionally, Duncan et al. (2018) found that any form of IPV victimization was associated with a higher total number of sexual partners, with IPV victims experiencing a 72% increase in receptive anal intercourse (RAI) partners (IRR = 1.72; 95% CI [1.10, 2.68]; *p* = 0.017) and an 80% increase in unprotected RAI partners (IRR = 1.80; 95% CI [1.03, 3.14]; *p* = 0.040).

## Discussion

This systematic review identified 35 studies examining the impacts of IPV among SGM individuals, covering a broad spectrum of sexual and gender identities and evaluating diverse types of IPV. The results revealed significant associations between IPV victimization and adverse mental health outcomes, including PTSD, depression, anxiety, suicidal ideation and suicide attempts, psychological distress, loneliness, and maladaptive coping styles. Additionally, IPV victimization was linked to negative physical health outcomes through risk behaviors, such as substance use, sexual behaviors, and sexually transmitted infections. These findings underscore the multifaceted impact of IPV on the overall well-being of SGM individuals.

### Intimate Partner Violence and Mental Health

This review identifies a robust correlation between IPV victimization and mental health symptomatology across diverse SGM identities. Specifically, findings from this review highlight links between IPV and PTSD in transgender individuals (Taber et al., 2023) and MSM (Stults et al., 2015), as well as associations between partner abuse and depression and anxiety among transgender and gender nonconforming adults (Henry et al., 2021). Additionally, lifetime IPV victimization has been strongly correlated with suicide attempts among transgender adults aged 50 years or older, underscoring the heightened vulnerability of this group.

Compounded identities further amplify these mental health risks. For instance, Bukowski et al. (2019) emphasize the elevated risk of depressive symptoms among Black transgender women, illustrating how overlapping marginalizations, such as race and gender identity, shape distinct mental health outcomes. Despite these important findings, a significant gap remains in our understanding of how the intersections of gender identity and sexual orientation influence mental health outcomes for IPV survivors. Addressing this gap through an intersectional lens is essential for capturing the unique ways race, ethnicity, sexual orientation, and gender identity converge to shape the IPV experiences of SGM survivors.

Furthermore, the literature indicates that maladaptive coping strategies in response to IPV victimization are prevalent among various SGM individuals, posing substantial risks for long-term psychological distress (Aldao et al., 2010; Sakulsriprasert et al., 2023). Understanding these dynamics across different identities is critical to psychosocial interventions that promote adaptive coping strategies, for instance, among SGM individuals.

In conclusion, the identified associations between IPV victimization and adverse mental health outcomes among SGM individuals underscore the necessity for culturally sensitive and inclusive interventions. Previous research (Murray et al., 2007; Rollè et al., 2018) reiterates the importance of recognizing and addressing the specific challenges faced by diverse SGM identities. Tailoring support to these needs is crucial for mitigating the detrimental impacts on mental health and promoting resilience and recovery among marginalized populations.

### Intimate Partner Violence, Risk Behaviors, and the Impacts on Physical Health

This systematic review underscores that IPV victimization's impact extends beyond mental health to encompass physical health concerns within SGM communities. Substance use, particularly in response to IPV exposure, has been well documented (Afifi et al., 2012; Cafferky et al., 2018). It is important to note that the relationship between IPV victimization and perpetration and substance use is exacerbated among SGM individuals due to the unique stressors they face (Meyer, 2003). Many SGM individuals may resort to substance use as a coping mechanism for managing the trauma and distress associated with IPV victimization (Augsburger & Elbert, 2017; Ben-Zur & Zeidner, 2009). Therefore, interventions should address these harmful coping mechanisms while also equipping individuals with healthier strategies.

Psychotherapy and psychoeducation programs specifically designed to address the unique experiences of individuals with minoritized identities could be invaluable for SGM survivors of IPV. These programs should not only focus on individual-level change but also incorporate community support and structural interventions to address discrimination, transphobia, stigma, and the broader systemic inequalities that perpetuate violence against SGM individuals.

Recognizing the impact of structural violence is essential for developing inclusive mental health services and community resources that acknowledge and respond to the unique needs of this population. Such an integrated approach can mitigate the immediate health risks associated with substance use and foster long-term well-being and resilience within this vulnerable population.

By combining individual, community, and structural interventions, we can create a comprehensive framework that effectively supports SGM individuals in navigating their experiences and promotes lasting change. This holistic perspective is critical for addressing the intersections of IPV, mental and physical health, and the systemic barriers that SGM individuals face.

In addition to substance use, the current study found risky sexual behaviors to be related to IPV victimization. These behaviors, including condomless sex, significantly increase the risk for HIV and STIs and are important to consider (Goesling et al., 2014). Interestingly, this study also found a significant association between PrEP use and IPV, particularly related to specific types of IPV, such as coercive control and emotional IPV. As shown in our study, STIs represent a critical aspect of the IPV–physical health nexus. IPV can increase the risk of STIs due to forced or non-consensual sexual activity, lack of control over sexual health decisions, or limited access to preventive measures. Recognizing this association emphasizes the importance of comprehensive sexual health support for SGM individuals experiencing IPV.

Healthcare providers should be equipped to address both the immediate health consequences and the broader implications of IPV on sexual health, ensuring that individuals receive appropriate care and resources. Furthermore, healthcare providers and clinics testing for HIV or STIs should start screening for IPV victimization, as many studies have found a significant relationship between both. This integrated approach can better support SGM individuals experiencing IPV, ensuring they receive not only appropriate sexual health care but also the necessary resources and interventions to address the broader implications of IPV on their well-being.

### Identity-Specific Intimate Partner Violence and Sexual and Gender Minority Individuals

In considering the experiences of SGM individuals, it is critical to recognize that not all SGM individuals experience the same levels of marginalization. In our review, we conceptualize SGM to encompass a wide range of non-cisgender and non-heterosexual identities and experiences, but it is important to highlight that SGM is not and should not be considered as a monolithic group.

We recognize that within the SGM category, individual identities intersect in unique ways, between sexual orientation, gender identity, race, ethnicity, socioeconomic status, and various others. The intersections of sexual identity, gender identity, and other identities (e.g., race, ethnicity, socioeconomic status) can lead to vastly different risks and experiences of IPV. For example, while cisgender gay white men primarily face heterosexism, they may not experience the compounded forms of oppression that transgender women, particularly transgender women of color, encounter.

Transgender individuals, especially those who are also racial and ethnic minorities, often face transphobia in addition to homophobia and racism. These intersecting oppressions can compound the risk of IPV, creating layers of vulnerability that single-axis analyses cannot fully capture. For instance, transgender women of color are subject to unique forms of structural violence, social exclusion, and discrimination that place them at heightened risk for both IPV and other forms of violence. It is thus essential to move beyond treating SGM individuals as a homogenous group and to account for the diversity of identities and oppressions that may interact to shape their experiences.

This review takes a first step at synthesizing existing literature on IPV among SGM populations. Findings from the current review underscore the critical importance of adopting an intersectionality lens to have a better understanding of the unique impacts of IPV faced by SGM individuals, particularly by focusing on their experiences rather than drawing a comparison between their cisgender heterosexual counterparts.

Taber et al. (2023) contributed significantly to this discourse by examining identity-specific IPV among transgender and gender nonconforming (TGNC) individuals (N = 200) who, in their majority (n = 175), had a minoritized sexual orientation (i.e., gay, lesbian, bisexual, pansexual, queer, or asexual). The authors utilized the T-IPV and IA scales to delve into experiences of IPV specific to survivors’ gender and sexual identities. The study’s findings revealed a positive association between T-IPV (e.g., IPV specific to the victim’s transgender identity) and identity abuse (e.g., IPV specific to the victim’s sexual orientation/gender identity) with multiple mental health outcomes, highlighting the intersecting vulnerabilities faced by TGNC and sexual minority individuals.

In addition, Scheer and Mereish (2021) explored the association between IPV and illicit substance use among sexual and gender minority youth (SGMY; *N* = 149), with a particular focus on identity abuse. Participants included gender (transgender women, transgender men, gender non-binary, and another non-cisgender identity; *n* = 63) and sexual (lesbian, gay, bisexual, queer, and other non-heterosexual identity; *n* = 147) minorities. The study’s findings demonstrated that transgender or gender non-binary SGMY were more likely to report identity abuse and physical abuse compared to cisgender SGMY. Furthermore, the authors also found that experiences of identity abuse were linked with higher rates of illicit substance use among SGMY, underscoring the intersecting vulnerabilities and experiences of different subgroups within SGM communities.

These studies collectively emphasize the need to move beyond a monolithic understanding of SGM populations and to consider the intersectionality of identities in the context of IPV and how such intersecting nature shapes individuals' experiences. Furthermore, the findings highlight the prevalence of identity-specific IPV within SGM communities. These experiences, combined with existing IPV-related impacts and the interaction of multiple minoritized identities with systems of oppression, place an additional burden on survivors (Meyer, 2003; Woulfe & Goodman 2020).

While our review primarily focused on gender identity and sexual orientation within SGM communities, it is important to note that some studies have examined the intersection of race/ethnicity with either gender identity or sexual orientation in the context of IPV. For instance, Beymer et al. (2017) explored the relationship between IPV and HIV among African-American MSM, while Bukowski et al. (2019) investigated IPV and depression among Black transgender women in the USA, and Wirtz et al. (2022) explored the association between IPV and STIs among black gay and bisexual men in the USA. Finally, Becerra et al. (2021) examined the experiences of IPV and mental health outcomes among Asian-American transgender adults in the USA. Notably, the latter study found that IPV victimization was substantially prevalent among the study participants and was associated with suicidal thoughts, suicidal attempts, and serious psychological distress. These studies highlight the intersecting vulnerabilities faced by SGM of color. Moving forward, there is a pressing need for future research to further explore and understand the complex intersections between gender identity, sexual orientation, and race/ethnicity in the context of IPV.

Overall, the current review recognizes the importance of addressing the unique experiences and challenges of SGM individuals, particularly by centering the research, prevention, and intervention efforts around those with marginalized identities. By doing so, we can develop more tailored support services that consider the unique needs of individuals across various SGM subgroups.

### Strengths and Limitations

The strengths of this systematic review lie in its comprehensive and rigorous approach to synthesizing existing research on IPV among SGM populations. The inclusion of a wide range of peer-reviewed studies from various disciplines and the systematic screening and data extraction processes enhance the review's credibility and reliability. Additionally, the review's focus on both mental and physical health outcomes provides a holistic understanding of the impact of IPV on SGM individuals. By highlighting associations between IPV victimization and adverse health outcomes, this study offers valuable insights that can inform future research and interventions aimed at addressing the unique needs of SGM individuals experiencing IPV. Lastly, the discussion of limitations and future research directions underscores the study's commitment to advancing knowledge and advocacy in this critical area of study.

Despite these strengths, it is crucial to acknowledge the limitations inherent in this study. A major limitation lies in the narrow focus on the intersection of sexual orientation and gender identity, without considering other intersecting identities, such as race, ethnicity, socioeconomic status, and education. Future studies should consider employing an intersectionality approach to their design and analysis to gain a better understanding of the complex interplay between such identities in the context of IPV. Additionally, the heterogeneity among the included studies in terms of sample characteristics, geographic locations, and measurement tools poses challenges in drawing universally applicable conclusions. Caution should be exercised when generalizing the findings to all SGM populations. Furthermore, the majority of studies in the review employed cross-sectional designs, which limit the ability to establish causal relationships between IPV victimization and the observed outcomes.

Longitudinal research is needed to better understand the temporal dynamics and long-term effects of IPV on the mental and physical health of SGM individuals. A notable gap in the literature is the limited representation of transgender, non-binary, and other gender nonconforming individuals. Future research should prioritize including these populations to gain a more comprehensive understanding of IPV experiences and outcomes within the broader SGM spectrum. Lastly, the included studies predominantly focused on Western contexts, potentially overlooking the unique experiences and cultural factors influencing IPV among SGM individuals in non-Western regions.

### Implications and Future Research Directions

The findings of this systematic review have significant implications for both research and practice. They unveil the impactful relationship between IPV and the mental and physical health of SGM individuals. Our findings emphasize the pressing need for integrated interventions that address both the mental and physical dimensions of health. It is clear that the trauma resulting from IPV can manifest in various ways, from PTSD and depression to substance use and risky sexual behaviors. To effectively support SGM survivors of IPV, it is imperative to recognize the interconnectedness of these outcomes and tailor interventions accordingly. Healthcare providers, social workers, and policymakers should be aware of the elevated risk of IPV-related physical and mental health consequences among SGM individuals, and services should be adapted to be inclusive and sensitive to their unique needs. Secondly, this review highlights the importance of comprehensive IPV screening protocols in healthcare settings to identify and support victims early on. Thirdly, our findings underscore the importance of adopting an intersectional lens, considering the diverse identities and stressors within the SGM community. Moving forward, targeted, evidence-based interventions and ongoing research are vital to ensuring the well-being and safety of SGM individuals, addressing the unique challenges they face, and promoting resilience within this vulnerable population.

Additionally, further research is warranted to delve deeper into the nuances of IPV within SGM communities. Longitudinal studies should be conducted to explore the causal relationships between IPV and health outcomes, shedding light on the mechanisms and pathways through which IPV exerts its negative effects. Additionally, qualitative research can help in capturing the lived experiences of SGM individuals, providing a richer understanding of the unique challenges they face and the resilience strategies they employ. Furthermore, interventions targeting IPV prevention and support among SGM populations should be rigorously evaluated for their effectiveness, considering both individual and structural factors.

Finally, as the landscape of gender and sexual identities continues to evolve, research must remain inclusive and adaptive to capture the experiences of emerging SGM groups, ensuring that the most vulnerable are not left invisible or underserved.

### Conclusion

In summary, this systematic review highlights the alarming impacts of IPV within SGM communities and its significant association with adverse physical and mental health outcomes. These findings not only demand immediate attention but also resonate as an urgent call to action. Healthcare providers, social workers, and policymakers must recognize the heightened risk of IPV-related health consequences among SGM individuals and adapt their services to be inclusive and sensitive. Comprehensive IPV screening protocols in healthcare settings are crucial for early identification and support. Furthermore, the review emphasizes the need for future research to explore causal relationships, capture lived experiences, evaluate interventions, and remain inclusive and adaptive in addressing emerging SGM identities. It is clear that there is limited existing research exploring the unique impacts IPV can have across SGM which has largely been overlooked in the literature as evidenced by our 35 included studies. Overall, addressing IPV within SGM communities is a critical imperative to ensure the well-being and safety of these individuals.

## Supplementary Information

Below is the link to the electronic supplementary material.

Supplementary file1 (DOCX 25 kb)

## Footnotes

1 SGM individuals are those who identify as lesbian, gay, bisexual, asexual, transgender, two-spirit, queer, intersex, those with same-sex or same-gender attractions or behaviors, those who do not self-identify with any labels but whose sexual orientation, gender identity or expression, or reproductive development is characterized by non-binary constructs of sexual orientation, gender, or sex assigned at birth (NIH, 2023)

2 The Medical Subject Headings (MeSH) thesaurus is a controlled and hierarchically organized vocabulary produced by the National Library of Medicine. It is used for indexing, cataloging, and searching of biomedical and health-related information. MeSH includes the subject headings appearing in MEDLINE/PubMed, the NLM Catalog, and other NLM databases (The National Library of Medicine).

## References

[CR1] Afifi, T. O., Henriksen, C. A., Asmundson, G. J. G., & Sareen, J. (2012). Victimization and perpetration of intimate partner violence and substance use disorders in a nationally representative sample. *Journal of Nervous and Mental Disease,**200*(8), 684–691. 10.1097/NMD.0b013e3182613f64 22850303 10.1097/NMD.0b013e3182613f64

[CR2] Aldao, A., Nolen-Hoeksema, S., & Schweizer, S. (2010). Emotion-regulation strategies across psychopathology: A meta-analytic review. *Clinical Psychology Review,**30*(2), 217–237. 10.1016/j.cpr.2009.11.004 20015584 10.1016/j.cpr.2009.11.004

[CR3] Augsburger, M., & Elbert, T. (2017). When do traumatic experiences alter risk-taking behavior? A machine learning analysis of reports from refugees. *PLoS ONE,**12*(5), e0177617. 10.1371/journal.pone.0177617 28498865 10.1371/journal.pone.0177617 PMC5428957

[CR4] Badenes-Ribera, L., Sánchez-Meca, J., & Longobardi, C. (2019). The relationship between internalized homophobia and intimate partner violence in same-sex relationships: A meta-analysis. *Trauma, Violence & Abuse,**20*(3), 331–343. 10.1177/1524838017708781 10.1177/1524838017708781 29333955

[CR5] Balsam, K., Rothblum, E., & Beauchaine, T. (2005). Victimization over the life span: A comparison of lesbian, gay, bisexual, and heterosexual siblings. *Journal of Consulting and Clinical Psychology,**73*, 477–487. 10.1037/0022-006X.73.3.477 15982145 10.1037/0022-006X.73.3.477

[CR6] Becerra, M. B., Rodriquez, E. J., Avina, R. M., & Becerra, B. J. (2021). Experiences of violence and mental health outcomes among Asian American transgender adults in the United States. *PLoS ONE,**16*(3), e0247812. 10.1371/journal.pone.0247812 33662045 10.1371/journal.pone.0247812 PMC7932064

[CR8] Ben-Zur, H., & Zeidner, M. (2009). Threat to life and risk-taking behaviors: A review of empirical findings and explanatory models. *Personality and Social Psychology Review,**13*(2), 109–128. 10.1177/1088868308330104 19193927 10.1177/1088868308330104

[CR9] Beymer, M. R., Harawa, N. T., Weiss, R. E., Shover, C. L., Toynes, B. R., Meanley, S., & Bolan, R. K. (2017). Are partner race and intimate partner violence associated with incident and newly diagnosed HIV infection in African-American men who have sex with men? *Journal of Urban Health,**94*(5), 666–675. 10.1007/s11524-017-0169-7 28616719 10.1007/s11524-017-0169-7 PMC5610124

[CR10] Bowleg, L. (2021). “The master’s tools will never dismantle the master’s house”: Ten critical lessons for Black and other health equity researchers of color. *Health Education & Behavior,**48*(3), 237–249. 10.1177/10901981211007402 34080476 10.1177/10901981211007402

[CR11] Braksmajer, A., Walters, S. M., Crean, H. F., Stephenson, R., & McMahon, J. M. (2019). Pre-exposure prophylaxis use among men who have sex with men experiencing partner violence. *AIDS and Behavior,**24*(8), 2299–2306. 10.1007/s10461-020-02789-2 10.1007/s10461-020-02789-2 PMC7857043 31953703

[CR12] Brooks, D., Wirtz, A. L., Celentano, D., Beyrer, C., Hailey-Fair, K., & Arrington-Sanders, R. (2021). Gaps in science and evidence-based interventions to respond to intimate partner violence among black gay and bisexual men in the U.S.: A call for an intersectional social justice approach. *Sexuality & Culture,**25*(1), 306–317. 10.1007/s12119-020-09769-7 33716496 10.1007/s12119-020-09769-7 PMC7946129

[CR13] Brooks, V. R. (1981). *Minority stress and lesbian women*. Lexington Books.

[CR14] Bukowski, L. A., Hampton, M. C., Escobar-Viera, C. G., Sang, J. M., Chandler, C. J., Henderson, E., Creasy, S. L., & Stall, R. D. (2019). Intimate partner violence and depression among black transgender women in the USA: The potential suppressive effect of perceived social support. *Journal of Urban Health,**96*(5), 760–771. 10.1007/s11524-019-00355-3 31037482 10.1007/s11524-019-00355-3 PMC6814667

[CR15] Burgess-Proctor, A. (2006). Intersections of race, class, gender, and crime: future directions for feminist criminology. *Feminist Criminology,**1*(1), 27–47. 10.1177/1557085105282899

[CR16] Cafferky, B. M., Mendez, M., Anderson, J. R., & Stith, S. M. (2018). Substance use and intimate partner violence: A meta-analytic review. *Psychology of Violence,**8*(1), 110–131. 10.1037/vio0000074

[CR18] Centers for Disease Control and Prevention. (2021, October 9). *Intimate Partner Violence*. Retrieved September 2, 2023

[CR19] Chen, W. T., Shiu, C., Yang, J. P., Chuang, P., Berg, K., Chen, L. C., & Chi, P. C. (2019). Tobacco, alcohol, drug use, and intimate partner violence among MSM living with HIV. *Journal of the Association of Nurses in AIDS Care: JANAC,**30*(6), 610–618. 10.1097/JNC.0000000000000090 31633629 10.1097/JNC.0000000000000090 PMC7609996

[CR21] Culbreth, R. E., Salazar, L. F., Spears, C. A., Crosby, R., Hayat, M. J., & Aycock, D. M. (2023). Stressors associated with tobacco use among trans women. *Transgender Health,**8*(3), 282–292. 10.1089/trgh.2020.0168 37342482 10.1089/trgh.2020.0168 PMC10277983

[CR22] Davis, D. A., Rock, A., Santa Luce, R., McNaughton-Reyes, L., & Barrington, C. (2022). Intimate partner violence victimization and mental health among men who have sex with men living with HIV in Guatemala. *Journal of Interpersonal Violence,**37*(3–4), NP1637–NP1657. 10.1177/0886260520928960 32552467 10.1177/0886260520928960 PMC7941092

[CR23] Devries, K. M., Mak, J. Y., Bacchus, L. J., Child, J. C., Falder, G., Petzold, M., Astbury, J., & Watts, C. H. (2013). Intimate partner violence and incident depressive symptoms and suicide attempts: A systematic review of longitudinal studies. *PLoS Medicine,**10*(5), e1001439. 10.1371/journal.pmed.1001439 23671407 10.1371/journal.pmed.1001439 PMC3646718

[CR24] Duncan, D. T., Goedel, W. C., Stults, C. B., Brady, W. J., Brooks, F. A., Blakely, J. S., & Hagen, D. (2018). A study of intimate partner violence, substance abuse, and sexual risk behaviors among gay, bisexual, and other men who have sex with men in a sample of geosocial-networking smartphone application users. *American Journal of Men’s Health,**12*(2), 292–301. 10.1177/1557988316631964 26873342 10.1177/1557988316631964 PMC5818104

[CR26] Edwards, K. M., Siller, L., Littleton, H., Wheeler, L., Chen, D., Sall, K., & Lim, S. (2021). Minority stress and sexual partner violence victimization and perpetration among LGBQ+ college students: The moderating roles of hazardous drinking and social support. *Psychology of Violence,**11*(5), 445–454. 10.1037/vio0000394

[CR29] Garcia-Moreno, C., Jansen, H. A., Ellsberg, M., Heise, L., & Watts, C. H. (2006). Prevalence of intimate partner violence: Findings from the WHO Multi-Country Study on Women’s Health and Domestic Violence. *Lancet,**368*(9543), 1260–1269. 10.1016/s0140-6736(06)69523-8 17027732 10.1016/S0140-6736(06)69523-8

[CR30] Goesling, B., Colman, S., Trenholm, C., Terzian, M., & Moore, K. (2014). Programs to reduce teen pregnancy, sexually transmitted infections, and associated sexual risk behaviors: A systematic review. *Journal of Adolescent Health,**54*(5), 499–507. 10.1016/j.jadohealth.2013.12.004 10.1016/j.jadohealth.2013.12.004 24525227

[CR32] Goldberg-Looney, L. D., Perrin, P. B., Snipes, D. J., & Calton, J. M. (2016). Coping styles used by sexual minority men who experience intimate partner violence. *Journal of Clinical Nursing,**25*(23–24), 3687–3696. 10.1111/jocn.13388 27192150 10.1111/jocn.13388 PMC5115985

[CR33] Guadalupe-Diaz, X. L., & Jasinski, J. (2017). “I wasn’t a priority, I wasn’t a victim”: Challenges in help seeking for transgender survivors of intimate partner violence. *Violence against Women,**23*(6), 772–792. 10.1177/1077801216650288 27271779 10.1177/1077801216650288

[CR34] Han, B., Wong, E. C., Mao, Z., Meredith, L. S., Cassells, A., & Tobin, J. N. (2016). Validation of a brief PTSD screener for underserved patients in federally qualified health centers. *General Hospital Psychiatry,**38*, 84–88. 10.1016/j.genhosppsych.2015.07.009 26386484 10.1016/j.genhosppsych.2015.07.009 PMC4698219

[CR35] Henry, R. S., Perrin, P. B., Coston, B. M., & Calton, J. M. (2021). Intimate partner violence and mental health among transgender/gender nonconforming adults. *Journal of Interpersonal Violence,**36*(7–8), 3374–3399. 10.1177/0886260518775148 29779457 10.1177/0886260518775148 PMC8463663

[CR36] Hillman, J. (2022). Lifetime prevalence of intimate partner violence and health-related outcomes among transgender adults aged 50 and older. *The Gerontologist,**62*(2), 212–222. 10.1093/geront/gnab067 34015135 10.1093/geront/gnab067

[CR37] Houston, E., & McKirnan, D. J. (2007). Intimate partner abuse among gay and bisexual men: Risk correlates and health outcomes. *Journal of Urban Health,**84*(5), 681–690. 10.1007/s11524-007-9188-0 17610158 10.1007/s11524-007-9188-0 PMC2231846

[CR42] McCown, C. M. (2023). Adverse childhood experiences and intimate partner violence in gender minority populations. *Graduate Theses, Dissertations, and Problem Reports*. 10.33915/etd.12148

[CR43] McDowell, M. J., Hughto, J. M. W., & Reisner, S. L. (2019). Risk and protective factors for mental health morbidity in a community sample of female-to-male trans-masculine adults. *BMC Psychiatry,**19*(1), 16. 10.1186/s12888-018-2008-0 30626372 10.1186/s12888-018-2008-0 PMC6327526

[CR44] Metheny, N., Tran, N. K., Scott, D., Dastur, Z., Lubensky, M. E., Lunn, M. R., Obedin-Maliver, J., & Flentje, A. (2024). Intimate partner violence is related to future alcohol use among a nationwide sample of LGBTQIA+ people: Results from The PRIDE Study. *Drug and Alcohol Dependence,**260*, 111342. 10.1016/j.drugalcdep.2024.111342 38820909 10.1016/j.drugalcdep.2024.111342 PMC12175093

[CR45] Meyer, I. H. (2003). Prejudice, social stress, and mental health in lesbian, gay, and bisexual populations: Conceptual issues and research evidence. *Psychological Bulletin,**129*(5), 674–697. 10.1037/0033-2909.129.5.674 12956539 10.1037/0033-2909.129.5.674 PMC2072932

[CR46] Miller, A. P., Wang, Y., Shoptaw, S., Gorbach, P. M., & Javanbakht, M. (2024). Substance use and associated experiences of intimate partner violence among MSM in Los Angeles, California. *Journal of Interpersonal Violence,**39*(13–14), 3088–3109. 10.1177/08862605231225517 38243744 10.1177/08862605231225517 PMC11126359

[CR47] Miltz, A. R., Lampe, F. C., Bacchus, L. J., McCormack, S., Dunn, D., White, E., Rodger, A., Phillips, A. N., Sherr, L., Clarke, A., McOwan, A., Sullivan, A., & Gafos, M. (2019). Intimate partner violence, depression, and sexual behaviour among gay, bisexual and other men who have sex with men in the PROUD Trial. *BMC Public Health,**19*(1), 431. 10.1186/s12889-019-6757-6 31023281 10.1186/s12889-019-6757-6 PMC6482482

[CR49] Murray, C. E., Mobley, A. K., Buford, A. P., & Seaman-DeJohn, M. M. (2007). Same-sex intimate partner violence. *Journal of LGBT Issues in Counseling,**1*(4), 7–30. 10.1300/J462v01n04_03

[CR50] National Institutes of Health. (2023). *Sexual & Gender Minority Research Office*.

[CR51] Page, M. J., McKenzie, J. E., Bossuyt, P. M., Boutron, I., Hoffmann, T. C., Mulrow, C. D., Shamseer, L., Tetzlaff, J. M., Akl, E. A., Brennan, S. E., Chou, R., Glanville, J., Grimshaw, J. M., Hróbjartsson, A., Lalu, M. M., Li, T., Loder, E. W., Mayo-Wilson, E., McDonald, S., & Moher, D. (2021). The PRISMA 2020 statement: An updated guideline for reporting systematic reviews. *British Medical Journal,**372*, n71. 10.1136/bmj.n71 33782057 10.1136/bmj.n71 PMC8005924

[CR53] Pantalone, D. W., Schneider, K. L., Valentine, S. E., & Simoni, J. M. (2012). Investigating partner abuse among HIV-positive men who have sex with men. *AIDS and Behavior,**16*(4), 1031–1043. 10.1007/s10461-011-0011-2 21822954 10.1007/s10461-011-0011-2 PMC4044042

[CR54] Passaro, R. C., Segura, E. R., Gonzales-Saavedra, W., Lake, J. E., Perez-Brumer, A., Shoptaw, S., Dilley, J., Cabello, R., & Clark, J. L. (2019). Sexual partnership-level correlates of intimate partner violence among men who have sex with men and transgender women in Lima, Peru. *Archives Sexual Behavior,**49*(7), 2703–2713. 10.1007/s10508-020-01682-2 10.1007/s10508-020-01682-2 PMC7494565 32270400

[CR56] Peitzmeier, S. M., Malik, M., Kattari, S. K., Marrow, E., Stephenson, R., Agénor, M., & Reisner, S. L. (2020). Intimate partner violence in transgender populations: Systematic review and meta-analysis of prevalence and correlates. *American Journal of Public Health,**110*(9), e1–e14. 10.2105/AJPH.2020.305774 32673114 10.2105/AJPH.2020.305774 PMC7427218

[CR57] Peitzmeier, S. M., Wirtz, A. L., Humes, E., Hughto, J. M. W., Cooney, E., & Reisner, S. L. (2021). The Transgender-Specific Intimate Partner Violence Scale for research and practice: Validation in a sample of transgender women. *Social Science and Medicine,**291*, 114495. 10.1016/j.socscimed.2021.114495 34710821 10.1016/j.socscimed.2021.114495 PMC8671347

[CR58] Peng, L., She, R., Gu, J., Hao, C., Hou, F., Wei, D., & Li, J. (2020). The mediating role of self-stigma and self-efficacy between intimate partner violence (IPV) victimization and depression among men who have sex with men in China. *BMC Public Health,**20*(1), 2. 10.1186/s12889-019-8125-y 31900234 10.1186/s12889-019-8125-y PMC6942407

[CR59] Reisner, S. L., Falb, K. L., Wagenen, A. V., Grasso, C., & Bradford, J. (2013). Sexual orientation disparities in substance misuse: The role of childhood abuse and intimate partner violence among patients in care at an urban community health center. *Substance Use and Misuse,**48*(3), 274–289. 10.3109/10826084.2012.755702 23368669 10.3109/10826084.2012.755702 PMC3918899

[CR60] Reuter, T. R., Newcomb, M. E., Whitton, S. W., & Mustanski, B. (2017). Intimate partner violence victimization in LGBT young adults: Demographic differences and associations with health behaviors. *Psychology of Violence,**7*(1), 101–109. 10.1037/vio0000031 28451465 10.1037/vio0000031 PMC5403162

[CR61] Rollè, L., Giardina, G., Caldarera, A. M., Gerino, E., & Brustia, P. (2018). When intimate partner violence meets same sex couples: A review of same sex intimate partner violence. *Frontiers in Psychology*, *9*, 1506. 10.3389/fpsyg.2018.01506 30186202 10.3389/fpsyg.2018.01506 PMC6113571

[CR62] Sabina, C., Cuevas, C., & Zadnik, E. (2014). Intimate partner violence among Latino women: Rates and cultural correlates. *Journal of Family Violence,**30*, 35–47. 10.1007/s10896-014-9652-z

[CR63] Sakulsriprasert, C., Thawornwutichat, R., Phukao, D., & Guadamuz, T. E. (2023). Early maladaptive schemas and addictive behaviours: A systematic review and meta-analysis. *Clinical Psychology & Psychotherapy*. 10.1002/cpp.2882 10.1002/cpp.2882 37464912

[CR64] Sarno, E. L., Newcomb, M. E., & Whitton, S. W. (2023). Minority stress and intimate partner violence among sexual and gender minorities assigned female at birth. *Psychology of Violence,**13*(3), 239–247. 10.1037/vio0000466 38045637 10.1037/vio0000466 PMC10691836

[CR65] Scheer, J. R., & Mereish, E. H. (2021). Intimate partner violence and illicit substance use among sexual and gender minority youth: The protective role of cognitive reappraisal. *Journal of Interpersonal Violence,**36*(21–22), 9956–9976. 10.1177/0886260519881001 31608738 10.1177/0886260519881001 PMC7153976

[CR66] Scheer, J. R., Woulfe, J. M., & Goodman, L. A. (2019). Psychometric validation of the Identity Abuse Scale among LGBTQ individuals. *Journal of Community Psychology,**47*(2), 371–384. 10.1002/jcop.22126 30207588 10.1002/jcop.22126 PMC6543831

[CR67] Silva-Martínez, E. (2017). “Allow me to speak”: Stories of ourage among immigrant Latina survivors of intimate partner violence. *Affilia,**32*(4), 446–460. 10.1177/0886109917721140

[CR70] Stephenson, R., & Finneran, C. (2017). Minority stress and intimate partner violence among gay and bisexual men in Atlanta. *American Journal of Men’s Health,**11*(4), 952–961. 10.1177/1557988316677506 27821702 10.1177/1557988316677506 PMC5675325

[CR74] Stults, C. B., Gao, S., Brandt, S. A., Taber, J. L., Lynn, S. G., Kaczetow, W., Lee, G., Cruise, A., & Krause, K. D. (2023). Intimate partner violence and mental health among transgender and gender diverse young adults. *Journal of Family Violence*. 10.1007/s10896-023-00579-7 37358980 10.1007/s10896-023-00579-7 PMC10220337

[CR75] Stults, C. B., Javdani, S., Greenbaum, C. A., Barton, S. C., Kapadia, F., & Halkitis, P. N. (2015). Intimate partner violence perpetration and victimization among YMSM: The P18 Cohort Study. *Psychology of Sexual Orientation and Gender Diversity,**2*(2), 152–158. 10.1037/sgd0000104 34859115 10.1037/sgd0000104 PMC8634533

[CR76] Subirana-Malaret, M., Gahagan, J., Parker, R., & Crowther-Dowey, C. (2019). Intersectionality and sex and gender-based analyses as promising approaches in addressing intimate partner violence treatment programs among LGBT couples: A scoping review. *Cogent Social Sciences*, *5*. 10.1080/23311886.2019.1644982

[CR78] Taber, J. L., Stults, C. B., Song, H., & Kaczetow, W. (2023). The role of internalized transphobia and negative expectations in the relationship between identity-specific intimate partner violence and mental health outcomes in transgender and gender nonconforming young adults. *Psychology of Sexual Orientation and Gender Diversity*. 10.1037/sgd0000641

[CR80] Veldhuis, C. B., Cascalheira, C. J., Delucio, K., Budge, S. L., Matsuno, E., Huynh, K., Puckett, J. A., Balsam, K. F., Velez, B. L., & Galupo, M. P. (2024). Sexual orientation and gender diversity research manuscript writing guide. *Psychology of Sexual Orientation and Gender Diversity,**11*(3), 365–396. 10.1037/sgd0000722 10.1037/sgd0000722 PMC12520195 41098880

[CR81] Whitton, S. W., Newcomb, M. E., Messinger, A. M., Byck, G., & Mustanski, B. (2019). A longitudinal study of IPV victimization among sexual minority youth. *Journal of Interpersonal Violence,**34*(5), 912–945. 10.1177/0886260516646093 27147275 10.1177/0886260516646093 PMC6538483

[CR82] Whitton, S. W., Swann, G., & Newcomb, M. E. (2024). Common and unique risk factors for intimate partner violence victimization among sexual and gender minority individuals assigned female at birth. *Violence and Victims*, *39*, 277-294. 10.1891/VV-2022-0125 39107073 10.1891/VV-2022-0125 PMC11781157

[CR83] Wirtz, A. L., Burns, P. A., Poteat, T., Malik, M., White, J. J., Brooks, D., Kasaie, P., & Beyrer, C. (2022). Abuse in the continua: HIV prevention and care outcomes and syndemic conditions associated with intimate partner violence among black gay and bisexual men in the southern United States. *AIDS and Behavior,**26*(11), 3761–3774. 10.1007/s10461-022-03705-6 35661018 10.1007/s10461-022-03705-6 PMC9561062

[CR84] Wong, C. F., Weiss, G., Ayala, G., & Kipke, M. D. (2010). Harassment, discrimination, violence, and illicit drug use among young men who have sex with men. *AIDS Education and Prevention*, *22*(4), 286–298. 10.1521/aeap.2010.22.4.286 20707690 10.1521/aeap.2010.22.4.286 PMC2962624

[CR85] Woulfe, J. M., & Goodman, L. A. (2020). Weaponized oppression: Identity abuse and mental health in the lesbian, gay, bisexual, transgender, and queer community. *Psychology of Violence,**10*(1), 100–109. 10.1037/vio0000251 10.1037/vio0000241 PMC7556696 33062388

[CR86] Xavier Hall, C. D., Newcomb, M. E., Dyar, C., & Mustanski, B. (2022). Patterns of polyvictimization predict stimulant use, alcohol and marijuana problems in a large cohort of sexual minority and gender minority youth assigned male at birth. *Psychology of Addictive Behaviors,**36*(2), 186–196. 10.1037/adb0000751 34081488 10.1037/adb0000751 PMC8639824

[CR87] Xu, L., Chang, R., Chen, Y., Xia, D., Xu, C., Yu, X., Chen, H., Wang, R., Liu, Y., Liu, S., Ge, X., Ma, T., Zhou, Y., Wang, Y., Ma, S., & Cai, Y. (2022). The prevalence of childhood sexual experiences and intimate partner violence among transgender women in China: Risk factors for lifetime suicidal ideation. *Frontiers in Public Health,**10*, 1037622. 10.3389/fpubh.2022.1037622 36755737 10.3389/fpubh.2022.1037622 PMC9900504

[CR88] Yu, Y., Cai, H., Chen, X., Xiao, F., Qin, K., & Li, J. (2023). Intimate partner violence and its associations among HIV-infected MSM with new drug abuse in Jinan, China. *BMC Public Health,**23*(1), 2517. 10.1186/s12889-023-17451-4 38102660 10.1186/s12889-023-17451-4 PMC10724906

[CR89] Zhu, Y., Hou, F., Chen, C., Wei, D., Peng, L., You, X., Gu, J., Hao, C., Hao, Y., & Li, J. (2021). Moderating effect of self-efficacy on the association of intimate partner violence with risky sexual behaviors among men who have sex with men in China. *BMC Infectious Diseases,**21*(1), 895. 10.1186/s12879-021-0661-2 34470607 10.1186/s12879-021-06618-2 PMC8408951

