# Supplementary material for: The Impact of Intimate Partner Violence on the Mental and Physical Health of Sexual and Gender Minorities: A Comprehensive Review of Quantitative Research
Source: Arch Sex Behav. 2024 Nov 4;54(2):433–47. doi: 10.1007/s10508-024-03023-z (PMC11836236; doi:10.1007/s10508-024-03023-z)
Supplement: Supplementary file 1 — Supplementary file1 (DOCX 25 kb) [file 10508_2024_3023_MOESM1_ESM.docx]

**Appendix S1.***MeSH terms used for systematic review search*

| **Search terms** | Search Platforms: PUBMED, APA PsycInFo, Google Scholar |
| --- | --- |
| 1  IPV | ("Intimate Partner Violence"[MeSH] OR "Domestic Violence"[MeSH] OR "Partner Violence, Intimate"[tiab] OR "Violence, Intimate Partner"[tiab] OR "Intimate Partner Abuse"[tiab] OR "Abuse, Intimate Partner"[tiab] OR "Partner Abuse, Intimate"[tiab] OR "Dating Violence"[tiab] OR "Violence, Dating"[tiab] OR intimate partner violence[tiab] OR domestic violence[tiab] OR IPV[tiab] OR partner abuse[tiab] OR spousal abuse[tiab]) |
| 2  SGM | ("Non-Heterosexual"[MeSH] OR "Non-Heterosexuals"[tiab] OR "Non Heterosexuals"[tiab] OR "Non-Heterosexual Person"[tiab] OR "Non-Heterosexual Persons"[tiab] OR "GLBT Person"[tiab] OR "GLBT Persons"[tiab] OR "Person, GLBT"[tiab] OR "Persons, GLBT"[tiab] OR "LGBTQ"[tiab] OR "Gender Diverse"[tiab] OR "Diverse, Gender"[tiab] OR "Gender Minority"[tiab] OR "Gender Minorities"[tiab] OR "Minorities, Gender"[tiab] OR "Minority, Gender"[tiab] OR "LBG Person"[tiab] OR "LBG Persons"[tiab] OR "Person, LBG"[tiab] OR "Persons, LBG"[tiab] OR "LGBT Person"[tiab] OR "LGBT Persons"[tiab] OR "Person, LGBT"[tiab] OR "Persons, LGBT"[tiab] OR "LGBTQ Person"[tiab] OR "LGBTQ Persons"[tiab] OR "Person, LGBTQ"[tiab] OR "Persons, LGBTQ"[tiab] OR "Sexual Minorities"[tiab] OR "Minorities, Sexual"[tiab] OR "Minority, Sexual"[tiab] OR "Sexual Minority"[tiab] OR "LGBTQIA"[tiab] OR "Bisexual"[tiab] OR "Bisexuals"[tiab] OR "Homosexual"[tiab] OR "Homosexuals"[tiab] OR "Queer"[tiab] OR "Queers"[tiab] OR "Gay"[tiab] OR "Gays"[tiab] OR "Lesbian"[tiab] OR "Lesbians"[tiab] OR "Men Who Have Sex With Men"[tiab] OR "Women Who Have Sex With Women"[tiab] OR "Transgender"[tiab]) |
| 3  IPV + SGM | ("Intimate Partner Violence"[MeSH Terms] OR "Domestic Violence"[MeSH Terms] OR "partner violence intimate"[Title/Abstract] OR "violence intimate partner"[Title/Abstract] OR "Intimate Partner Abuse"[Title/Abstract] OR "abuse intimate partner"[Title/Abstract] OR "Dating Violence"[Title/Abstract] OR "violence dating"[Title/Abstract] OR "Intimate Partner Violence"[Title/Abstract] OR "Domestic Violence"[Title/Abstract] OR "IPV"[Title/Abstract] OR "partner abuse"[Title/Abstract] OR "spousal abuse"[Title/Abstract]) AND ("non heterosexuals"[Title/Abstract] OR "non heterosexuals"[Title/Abstract] OR "Non-Heterosexual Persons"[Title/Abstract] OR "GLBT Person"[Title/Abstract] OR "GLBT Persons"[Title/Abstract] OR "LGBTQ"[Title/Abstract] OR "Gender Diverse"[Title/Abstract] OR "diverse gender"[Title/Abstract] OR "Gender Minority"[Title/Abstract] OR "Gender Minorities"[Title/Abstract] OR "minorities gender"[Title/Abstract] OR "minority gender"[Title/Abstract] OR "LBG Persons"[Title/Abstract] OR "LGBT Person"[Title/Abstract] OR "LGBT Persons"[Title/Abstract] OR "persons lgbt"[Title/Abstract] OR "LGBTQ Person"[Title/Abstract] OR "LGBTQ Persons"[Title/Abstract] OR "person lgbtq"[Title/Abstract] OR "persons lgbtq"[Title/Abstract] OR "Sexual Minorities"[Title/Abstract] OR "minorities sexual"[Title/Abstract] OR "minority sexual"[Title/Abstract] OR "Sexual Minority"[Title/Abstract] OR "LGBTQIA"[Title/Abstract] OR "Bisexual"[Title/Abstract] OR "Bisexuals"[Title/Abstract] OR "Homosexual"[Title/Abstract] OR "Homosexuals"[Title/Abstract] OR "Queer"[Title/Abstract] OR "Queers"[Title/Abstract] OR "Gay"[Title/Abstract] OR "Gays"[Title/Abstract] OR "Lesbian"[Title/Abstract] OR "Lesbians"[Title/Abstract] OR "Men Who Have Sex With Men"[Title/Abstract] OR "Women Who Have Sex With Women"[Title/Abstract] OR "Transgender"[Title/Abstract]) |

**Appendix S2.**

*Summary of Systematic Review Findings*

| **Citation** | **Sample Size** | **Sexual orientation** | **Gender Identity** | **IPV Measures** | **Mental Health measures** | **Physical Health measures** |
| --- | --- | --- | --- | --- | --- | --- |
| Taber et al. (2023) | 200 | Gay, lesbian, bisexual, pansexual, queer, asexual, straight, other non-heterosexual orientation | Transgender men, transgender women, nonbinary, genderqueer, gender fluid, gender nonconforming, agender, two-spirit | Transgender-related Intimate Partner Violence scale and the Identity Abuse scale | Posttraumatic Stress Disorder Checklist (PCL-6), General Anxiety Disorder Scale (GAD-7), PHQ-9 | N/A |
| Edwards et al. (2021) | 1,221 | Bisexual, pansexual, gay, queer, questioning, demisexual, other non-heterosexual orientation | Women, men, genderqueer, gender non-conforming, nonbinary, transgender identity | Sexual and Gender Minority Conflict Tactics Scale 2 (SGM-CTS-2) | N/A | The 5-item Alcohol Use Disorders Inventory |
| Woulfe & Goodman (2020) | 734 | Bisexual, gay, lesbian, queer, pansexual | Men, women, transgender, nonbinary | The Identity Abuse Scale, The Conflict Tactics Scale (CTS-2), The Psychological Maltreatment of Women Inventory | The PTSD Checklist Civilian Version (PCL-C), The Center for Epidemiological Studies Depression Scale Revised (CESD-R) | N/A |
| Stults et al. (2015) | 528 | Homosexual and heterosexual | N/A | “Yes/no” questions | The Trauma Awareness and Treatment Center (TACT) Post-traumatic Stress Questionnaire, The Beck Anxiety Inventory (BDI) | N/A |
| Reuter et al. (2017) | 172 | Lesbian, gay bisexual, questioning, other non-heterosexual orientation | Male, female, male-to-female transgender, female-to-male transgender | HIV-Risk Assessment of Sexual Partnerships (H-RASP) | The Brief Symptom Inventory (BSI-18) | The HIV-Assessment of Sexual Partnerships (H-RASP), “yes/no” questions about substance use |
| Whitton et al. (2019) | 248 | Gay, lesbian, bisexual, questioning, other non-heterosexual orientation | Cisgender, male-to-female transgender, female-to-male transgender | HIV-Risk Assessment for Sexual Partners (H-RASP) | The Brief Symptom Inventory (BSI-18) | The HIV-Assessment of Sexual Partnerships (H-RASP), “Yes/no” questions about substance use |
| McDowell et al. (2019) | 150 | N/A | Masculine spectrum identity (female-to-male and/or trans-masculine), non-binary, binary | “Yes/no” Questions | PC-PTSD, The Brief Symptom Inventory (BSI-18) | N/A |
| Bukowski et al. (2019) | 493 | Transgender women who have sex with men | Female, transgender, or having transitioned from male to female gender | “Yes/no questions” | The Center for Epidemiologic Studies Depression (CES-D 10) | N/A |
| Xu et al. (2023) | 247 | Homosexual, heterosexual, bisexual | Transgender women | Transgender Youth Project Questionnaire | “Yes/no” question to assess lifetime suicidal ideation | N/A |
| Reisner et al. (2013) | 2,653 | Bisexual, gay, lesbian, other non-heterosexual orientation, heterosexual | Non-transgender identity | “Yes/no” questions | N/A | “Yes/no” questions about lifetime substance use |
| Henry et al. (2021) | 78 | Gay, lesbian, bisexual, queer, other non-heterosexual orientation, heterosexual | Transgender men, transgender women, gender non-conforming, another non-cisgender identity | Revised Conflict Tactics Scale Short-Form | The Hopkins Symptoms Checklist-25 | N/A |
| Becerra et al. (2021) | 27,715 | N/A | Transgender individuals | “Yes/no questions” | “Yes/no” questions about suicidal ideation and attempts | N/A |
| Braksmajer et al. (2020) | 663 | Men Who Have Sex With Men | N/A | IPV-GBM Scale | N/A | “Yes/no” questions about PrEP use |
| Zhu et al. (2021) | 578 | Men Who Have Sex With Men | N/A | IPV-GBM Scale | N/A | “Yes/no” questions about risky sexual behaviors |
| Xavier Hall et al. (2022) | 1,202 | Gay, bisexual, other sexual orientations | Cisgender male and gender minorities | “Yes/no” questions | N/A | The Alcohol Use Disorder Identification Test (AUDIT), “yes/no” questions about substance use |
| Beymer et al. (2017) | 1,974 | Gay, bisexual, Men Who Have Sex With Men, men who have sex with transgender persons | N/A | “Yes/no” questions | N/A | 82-item risk assessment questionnaire |
| Goldberg-Looney et al. (2016) | 89 | Gay, bisexual. Queer, other non-heterosexual orientation | Men | Revised Conflict Tactics Scale, Short-Form | The Brief COPE Scale | N/A |
| Passaro et al. (2020) | 576 | Bisexual, homosexual, heterosexual | Transgender | “Yes/no” questions | N/A | The Alcohol Use Disorder Identification Test (AUDIT-10), “yes/no” questions about substance use, “yes/no” questions about risky sexual behaviors |
| Wirtz et al. (2022) | 629 | Gay, same-gender loving, bisexual, other non-heterosexual orientation | N/A | “Yes/no questions” | The Patient Health Questionnaire-2 scale | 3-item AUDIT-C Scale, and “yes/no” questions about risky sexual behaviors |
| Peitzmeier et al. (2021) | 661 | N/A | Female or Woman, Transwoman, nonbinary, another gender identity | T-IPV, WHO multi-country study questionnaire, “Yes/no” questions, | PC-PTSD, and the Kessler-6 | AUDIT-C, DAST-10, “yes/no” questions about risky sexual behaviors |
| Miltz et al. (2019) | 436 | Gay, bisexual, Men Who Have Sex With Men | N/A | “Yes/no” questions | PHQ-9 | “Yes/no” questions about substance use and risky sexual behaviors. |
| Davis et al. (2022) | 374 | Gay, bisexual, and other Men Who Have Sex With Men | N/A | “Yes/no” questions” | 2-item Generalized Anxiety Disorder Assessment (GAD-2), and the 2-item Patient Health Questionnaire (PHQ-2) | The 11-item DSM-5 Alcohol Use Disorder (AUD) assessment |
| Scheer & Mereish (2021) | 149 | Lesbian, gay, bisexual, queer, other non-heterosexual orientation | Cisgender women, cisgender men, transgender women, transgender men, gender nonbinary | The Identity Abuse Scale, Conflict Tactics Scale, Short form, Psychological Maltreatment of Women Inventory | N/A | “Yes/no” questions about substance use |
| Chen et al. (2020) | 120 | Men Who Have Sex With Men | N/A | 15-item Danger Assessment Scale | 10-item anxiety subscale derived from the Symptom Checklist-90-R | Modified version of the 10-item Substance Use Assessment |
| Miller et al. (2024) | 557 | Men Who Have Sex With Men | N/A | Two items from the National HIV Behavioral Surveillance (NHBS) questionnaire | Center for Epidemiologic Studies Depression scale [CES-D] | Self-reported substance use in the past 6 months  Self-report of PrEP use |
| Pantalone et al. (2012) | 168 | Men Who Have Sex With Men | N/A | The Revised Conflict Tactics Scale (CTS2) | 10-item state anxiety subscale of the State-Trait Personality Inventory (STPI)  The Center for Epidemiological Study-Depression Scale (CES-D  Passive Suicidal Behavior subscale of the Harkavy Asnis Suicide Survey (HASS)  The Brief COPE | Daily Drug-Taking Questionnaire (DDTQ)  Self-reported HIV medication adherence |
| Wong et al. (2010) | 526 | Men Who Have Sex With Men | N/A | 12 -item adapted scale to measure physical, sexual, emotional abuse | N/A | Self-reported illicit drug use |
| Yu et al. (2023) | 294 | Men Who Have Sex With Men | N/A | Dating Violence Questionnaire (DVQ | Patient Health Questionnaire (PHQ-2  Generalized Anxiety Disorder (GAD-2)  Beck Scale for Suicide Ideation-Chinese Version (BSI-CV) | Self-reported drug use in the past 6 months  Self-report sexual risk behavior and ART medication adherence |
| Stults et al. (2023) | 200 | Queer, Pansexual, Gay, Lesbian, Bisexual, Straight, Other | Genderqueer, gender fluid, gender non-conforming, agender, nonbinary, female, male | modified version of the Conflict Tactics Scale, Transgender-related Intimate Partner Violence Scale (T-IPV, The Identity Abuse Scale | Patient Health Questionnaire (PHQ-9) e General Anxiety Disorder scale (GAD-7)  Post-traumatic Stress Disorder Checklist (PCL-6 | N/A |
| Peng et al. (2020) | 578 | Men Who Have Sex With Men | N/A | IPV-GBM | The Center for Epidemiologic Studies Depression 10 (CES-D-10) | N/A |
| Metheny et al. (2024) | 3,783 | Asexual, bisexual, gay, lesbian, pansexual, queer, questioning, same-gender loving, heterosexual, two-spirit, another sexual orientation | Agender, cisgender man, cisgender woman, genderqueer, man, non-binary, questioning, transgender man, transgender woman, two-spirit, woman, another gender identity | Extended-Hurt, Insulted, Threaten, Scream (E-HITS) | N/A | The Alcohol Use Disorders Identification Test (AUDIT) |
| Houston & McKirnan (2007) | 817 | Men Who Have Sex With Men | N/A | “Yes/no” questions | Center for Epidemiological Studies’ Depression Scale (CES-D | Self-reported drug and alcohol use |
| Hillman (2022) | 3,462 | Lesbian, Gay, Bisexual, Heterosexual, Asexual, Other | Female/MTF/trans-female,  Male/FTM/trans-male, Nonbinary/genderqueer | Used 25 items from the National Intimate Partner Violence and Sexual Violence Survey | K6 Non-Specific Distress Scale  Self-reported suicidal ideation/attempts | Items from the National Survey on Drug Use and Health  Self-reported alcohol and drug use |
| Duncan et al. (2018) | 175 | Gay, Bisexual, Other Men Who Have Sex With Men | N/A | “Yes/no” questions | N/A | Self-reported substance use  Self-reported sexual risk behaviors |
| Culbreth et al. (2023) | 162 | N/A | Female, transgender person, or other non-cisgender identities | “Yes/no” questions | N/A | Self-reported tobacco use |
